# Utilization of Antibacterial Nanoparticles in Photocurable Additive Manufacturing of Advanced Composites for Improved Public Health

**DOI:** 10.3390/polym13162616

**Published:** 2021-08-06

**Authors:** Christopher Billings, Changjie Cai, Yingtao Liu

**Affiliations:** 1 School of Aerospace and Mechanical Engineering, University of Oklahoma, Norman, OK 73019, USA; christopherbillings@ou.edu; 2 Department of Occupational and Environmental Health, University of Oklahoma Health Sciences Center, University of Oklahoma, Oklahoma City, OK 73104, USA

**Keywords:** additive manufacturing, photopolymerization, digital light processing, nanoparticles, antimicrobial, public health

## Abstract

This paper presents the additive manufacturing and characterization of nanoparticle-reinforced photocurable resin-based nanocomposites with a potential antimicrobial function for improved public health applications. Two types of photocurable resins are reinforced by titanium dioxide (TiO_2_) or zinc oxide (ZnO) nanoparticles with average diameters in the 10–30 nm range to provide antimicrobial properties. The developed nanocomposites can be additively manufactured using the digital light processing method with an outstanding surface quality and precise geometrical accuracy. Experimental characterizations are conducted to investigate key mechanical properties of the 3D printed nanocomposites, including Young’s Modulus, tensile strength, and abrasion resistance. Specimens produced were observed to demonstrate the following characteristics during testing. Tensile strength increased by 42.2% at a maximum value of 29.53 MPa. The modulus of elasticity increased by 14.3%, and abrasion resistance increased by 15.8%. The proper dispersion of the nanoparticles within the cured resin is validated by scanning electron images. The wettability and water absorption testing results indicate that the developed nanocomposites have an outstanding water resistance capability. The pairing of digital light processing with these novel nanocomposites allows for the creation of complex composite geometries that are not capable through other manufacturing processes. Therefore, they have the potential for long-term usage to improve general public health with antimicrobial functionality. The pairing of an unmodified photocurable resin with a 1% ZnO concentration demonstrated the most promise for commercial applications.

## 1. Introduction

Additive manufacturing (AM) has played a significant role in polymer and composite manufacturing for broad biomedical and public health applications in recent years [1,2,3,4]. Due to the broad array of AM techniques, such as fused deposition modeling and direct ink writing, traditional biocompatible polymers and composites can be rapidly converted into novel devices and products to enhance medical treatment and public health [5]. Additionally, the integration of AM technologies with novel polymers and nanomaterials with beneficial functionalities has opened new directions for developing cutting-edge devices, sensors, and tools that may revolutionize medical treatment and prevent infectious diseases.

Photocurable-based AM is a unique manufacturing option due to the high product quality and low processing temperature. These types of AM systems are usually referred to as stereolithography (SLA) and digital light processing (DLP), and use an ultraviolet light source to cure photopolymers layer by layer with a high spatial resolution and surface quality [6,7]. This feature allows the photopolymerization-based AM systems to create parts with almost perfect microstructures, which is rare for other AM processes, such as fused deposition modeling. The build plate is the only moving part in these machines traveling in the Z direction. This mechanically simple design allows for a precise process control and optimization during AM for various photocurable polymers and nanoparticle-reinforced composites.

Wear resistance, durability, and water resistance of polymers and nanocomposites are critical in healthcare environment applications. For example, *Clostridioides difficile* (*C. difficile*) infection (CDI) is the leading cause of nosocomial diarrhea worldwide with substantial morbidity, mortality, and healthcare cost [8,9]. In addition, the Centers for Disease Control and Prevention (CDC) has classified *C. difficile* as an “urgent” threat (the highest threat) in its antimicrobial resistance threat report [10]. Contact precautions and environmental hygiene have been only moderately successful for CDI control, which remains a seemingly intractable problem. The challenging is that the *C. difficile* spores that become airborne during patient care activities travel long distances, contaminating environmental surfaces remote from the sources [11]. Thus, CDI is related to many healthcare activities, including the bed making, bedpan washing, provider movement, housekeeping activities, and toilet flushing [11,12,13]. The *C. difficile* bacteria can grow and be emitted from both wet (such as flushing toilet bowl water and bowl surfaces) and dry (such as table, ground, and wall surfaces) environments. Therefore, it is critical to test the key mechanical properties of the manufactured composites to prevent the bacteria growth and, then, to reduce their emissions.

Nanoparticles (particle diameter < 100 nm), such as titanium dioxide (TiO_2_) and zinc oxide (ZnO), have been widely studied due to their significant antimicrobial potentials, especially valuable to antibiotic-resistant bacteria [14,15]. For example, when *Escherichia coli* (*E. coli*) colonies were exposed to surfaces treated with nanoparticles, prevention in further bacteria growth was observed [16]. The nanoparticle antimicrobial capacities can be further enhanced by ultraviolet (UV) illumination due to the photocatalysis effects of these nanoparticles [17,18]. UV illumination was observed to be a vital mechanism in activating the antibacterial properties of the given nanoparticles [19,20,21,22]. The size-controlled nanoparticles can also improve the antibacterial efficacy because the smaller sizes can easily penetrate into bacterial membranes due to their larger particle surface areas [23,24]. If exposed to light in the UV spectrum, the nanoparticles became safe for use in the human body.

Testing of these nanoparticles relies on the test surface having a light dispersion of the nanoparticle to work. To achieve these traits in a polymer application, it would be required for the part to have a continuous surface coating of the nanoparticle on all surfaces. This coating would have to either be wear-resistant or rely on a mechanism that ensures new nanoparticles are exposed to the surface during wear. In addition, proper particle dispersion would have to be investigated through a scanning electron microscope (SEM). Once the part’s surface is successfully coated with the nanoparticle, antibacterial traits can be expected to be seen. 

A solution to the above issue is to create a nanocomposite polymer. This nanocomposite would have to show a proper dispersion throughout the entirety of the part and have a large enough concentration of the selected nanoparticle to be effective. A strong candidate for the matrix material would be a photocurable resin due to the material’s workability before activation. Ensuring the viscosity of the matrix is low enough that the addition of the nanoparticle will not hinder use case performance is less of a concern when using liquid resins [25]. Using proper mixing methods and quick curing will allow for a homogenous nanocomposite that would not only show nanoparticles on the surface but would continuously expose new nanoparticles as the material degrades over time.

Using photocurable-based AM processes with a nanocomposite resin as suggested above, would allow for a complex model creation, high-speed production, and antibacterial traits. Utilizing LCD-based curing technology would allow for entire layers to be cured simultaneously to ensure the chemical reaction of the curing polymer would not disturb the surrounding nanocomposite mixture. Nanoparticles such as TiO_2_ have been successfully used in AM techniques to manufacture nanocomposite structures [26]. The inclusion of ZnO will allow for a direct comparison of two different nanoparticles that both inhibit bacterial growth and their effect on the AM process.

This research addresses the high cost and complexity associated with producing composites using traditional methods by creating a novel antibacterial nanocomposite designed for use in digital light processing machines. Utilizing two different proven antibacterial nanoparticles and two different matrix materials, researchers explore the physical properties of the novel nanocomposites and their effect on the manufacturing process. These nanoparticles are chosen due to their already current use in nanofilms and tools found in the healthcare industry [27,28,29]. These nanocomposites paired with AM allow for creating specialty parts and tools vital to the healthcare industry.

## 2. Materials and Methods

### 2.1. Equipment Used

The DLP-based polymer 3D printer used to conduct this study was an Anycubic Photon (Anycubic, Commerce, CA, USA) with a build volume of 115 mm × 65 mm × 155 mm. This printer has a *y*-axis resolution of 1.25 μ and a *z*-axis resolution of 25 μ. The printer utilizes an LCD panel to mask off LEDs that emit light in the 405 nm wavelength. No physical modifications to the printer were performed. The two resins that were used were produced by ELEGOO Mars (Elegoo, Shenzhen, China) and were a standard LCD UV-curing photopolymer rapid resin and an ABS-like LCD UV-curing photopolymer rapid resin. The two nanoparticles selected were TiO_2_ and ZnO in the 10–30 nm range from Sky Spring Nanomaterials (Sky Spring, Houston, TX, USA). For tensile testing, an Instron 5969 (Instron, Norwood, MA, USA) was used with a 5 kN load cell. Abrasion tests were completed using a LaboPol-5 (Struers Cleveland, OH, USA) with a LaboForce-3 head attachment. A grinding disc with aluminum oxide abrasive sized at 50 μ, or 240 grit pad, was used as the grinding medium.

### 2.2. Nanocomposite Synthesis Requirements

To synthesize the nanocomposite resin, several steps were followed to help increase printability. Initially, the ability for the nanocomposite to cure required testing as the addition of a non-curable agent led to reduced bonding and light transmission throughout the print. The software used for preparing prints, CHITUBOX, allowed for modification of all printing steps. The criteria used to determine successful prints included the ability for the print to reliably adhere to the build plate and for the printer to have a minimal loss in part resolution. The addition of the nanoparticle to the resin created two issues. The first issue was that as the nanoparticle percentage increased, the viscosity of the novel composite also increased. This directly affected the printer’s ability to reset between layers and allowed for an even nanoparticle dispersion. The second issue was that as the nanoparticle percentage increased, the ability for the printer to cure each layer adequately decreased. The modified parameters were the number of initial layers, the initial layer cure time, the remaining layer cure time, and the initial z distance. The first issue was addressed using different concentrations of nanoparticles to test for proper return flow into the recess created due to the curing part. As the printer finished a layer, the build plate rose above, releasing the part from the FEP sheet to allow the new resin to flow into the recess. The time could be adjusted through software but was left constant to keep print times at a reasonable speed. Increasing this time would also create issues with particle settlement. Results showed that concentrations up to 5% could adequately fill the void in the time provided. To address the second issue of reducing bonding between the resin and the build plate, the initial z height was decreased to create a thinner layer of composite between the FEP sheet and the aluminum build plate. This helped ensure that the resin was forced into the micro-abrasions on the build plate, therefore, increasing build plate adhesion. This also reduced the amount of resin that required curing on the first layer.

Along with this change, the initial layer curing time was doubled from 60 s to 120 s. An increased cure time would help improve the consistency of the cure across the layers. A total of 5 bottom layers were used for the prints. Lastly, the cure time for the remaining layers was increased from 8 s to 15 s. With these settings in place, varying concentrations of nanoparticles were added to the composite for testing. Adequate bonding and detail retention were easily held at a 1% nanoparticle addition. At around 2.5% addition, failed prints began to arise due to a failure in the bonding of the first layer.

**Figure 1:**
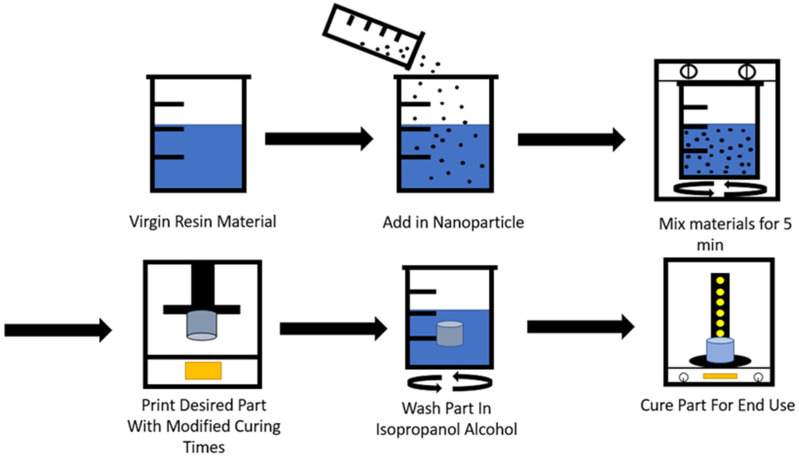
Complete manufacturing process.

There were several different mixing methods employed to introduce the nanoparticles to the matrix resins. First was a process of hand mixing the two components for five minutes with a stir rod. The second was to shake the mixture in a closed bottle until no visible particles could be identified. The last method was to use a stirring plate. Hand mixing was an effective procedure but required careful inspection of the mixture as it is poured into the printer to ensure the mixture is homogenous without the formation of clumps. Filters with pore diameters of less than 1 mm could be employed to help ensure no large particles were introduced into the printing vat, leading to print failures. The second method provided a greater degree of homogeneity throughout the mixture due to the forces involved in the mixing process and the ability to change the direction of the fluid flow within the mixing chamber. The last method provided similar results to the first as the stirring plate would often have difficulty mixing the resin at speeds above 100 rpm due to the high viscosity of the mixture. The resins mixed in the enclosed bottle also showed less particle settlement. A proper mixture would suspend particles for up to 6 h of printing without needing to be remixed. Therefore, an enclosed centrifugal mixer is recommended as the best machine for synthesizing the nanocomposite resin. The entire manufacturing process for the resin and part is depicted in Figure 1.

### 2.3. Addressing Particle Settlement

Other essential concerns with the use of a non-homogenous nanocomposite included particle settlement over time. As the only motion during the print was the build plate repeatedly raising and lowering, the nanoparticles would begin to settle over time as their density was higher than that of the resin matrix. This issue was most noticeable at higher particle concentrations and would cause layer adhesion issues during the print as the particles settled and increased the relative concentration at that layer. There were several approaches used to remedy this issue. The first approach was to start with a minimal amount of nanoparticle addition, as previously discussed, as this would help ensure proper distribution throughout the matrix material. The second and more proactive step was to pause the print on the hour and remix the composite by hand. This was performed in the printer with a plastic scraper as not to damage the FEP sheet. Finally, removing the resin vat could cause alignment issues with the printer and, therefore, the mixing was performed inside the vat with the build plate raised to the maximum Z height.

### 2.4. Post Processing

After removal from the build plate, all parts were put into an isopropyl alcohol bath on a stirring plate for a minimum of 6 minutes. This allowed for the isopropyl alcohol to dissolve any remaining uncured resin on the parts, which is critical to retaining high part resolution during the final stage. After a thorough cleaning, the part was put into a UV chamber for a minimum of 4 min. Again, time was added based on the appearance of the part until a flat matte sheen was achieved.

### 2.5. Part Selection

**Figure 2:**
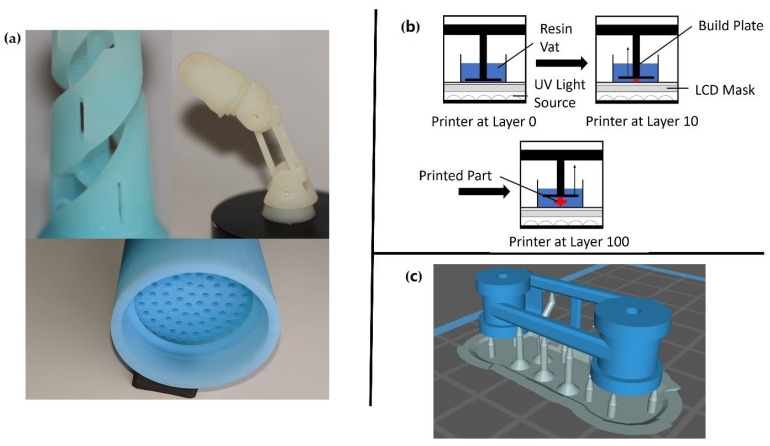
(**a**) Three-dimensional printed example pieces included a chess rook, a prosthetic finger, and a pipe fitting. (**b**) The manufacturing process for LCD-based printing systems. (**c**) Middle finger joint in blue and support material in grey.

To demonstrate the 3D printability of the novel nanocomposite, several different complex parts were printed and assembled. The first of the three primary tests that were conducted consisted of a chess rook with intricate detail to observe the nanocomposite resin’s ability to maintain surface details and internal geometries within a single part. Secondly, a large pipe fitting was printed to show the material’s ability to be used in fluid flow systems successfully. Lastly, a prosthetic finger was printed to give a direct example of a biomedical application. All these parts can be seen in Figure 2a. Along with these prints, over 30 tensile test specimens were printed.

The pipe fitting was printed to showcase the part’s ability to be used in a fluid flow system. As discussed later, the low water absorption of these novel resins over time allowed for part adaptation into fluid environments where antibacterial properties were beneficial. Many parts of the healthcare industry are often in contact with different types of fluids and must be meticulously cleaned as a moist environment breeds bacteria. The fittings successfully printed here showcased the ability to produce complex part geometries that would help lead to less turbulent flow within a system. This would be incredibly beneficial when working with fluid systems where turbulent flow that traps air bubbles is undesirable. Additionally, the nature of the material would lead to fewer concerns for contamination and longer implementation cycles before cleaning or replacement. As these parts can be produced in mere hours for a fraction of the cost of most medical equipment, the viability of this technology in medical environments increases.

Lastly, a working prosthetic finger designed by Danger Creations was printed to show the opportunities this technology provides. The part has many intricate details and moving parts that perfectly interface together. The prosthetic finger can be manufactured in under 2 h. This technology allows for weight optimization of medical apparatuses that are not possible with other manufacturing techniques. Since weight is a critical factor in user experience and comfort, customizable options such as these are consumer-oriented. Because the novel nanocomposite was developed explicitly for use in digital light processing machines, the design freedom given to medical engineers was greatly expanded. In addition, the material cost for this finger was just 18 cents for the nanoparticles and resin material. Given a uniform dispersion, a low concentration of nanoparticles led to a low-cost nanocomposite. The pairing of a low-cost nanocomposite with AM technology provides the best solution for corporations needing intricate one-off parts. Due to the elimination of expensive machinery and custom molding, this technology offers an unparalleled cost advantage for the healthcare industry. This drastic reduction in cost and manufacturing time will revolutionize the medical industry if implemented on a per hospital level, all while delivering better care.

### 2.6. Tensile Testing

Tensile testing was performed using the ASTM bar-type IV standard to verify uniformity between prints and the effect of the nanoparticle on the physical characteristics of the base resin materials. The specimens were 70 mm in length as this maximized the build plate area that was available. In addition, this was performed so that it would show up as lower tensile stress values if there were nonuniformity throughout the material.

## 3. Results

### 3.1. Nanoparticle Dispersion Methods

Extensive testing was performed to produce complex geometries and parts that could not be traditionally manufactured using subtractive or casting methods. Within this scope, special attention was given to the printer’s ability to hold tolerances and detail resolution throughout the entirety of the print. As this technology could be implemented in the biomedical field, high-speed fabrication and low failure rates are critical to widespread adoption.

#### 3.1.1. Comparing the Matrix Materials

The first dataset analyzed was the difference in tensile strength of the ABS-like resin and the control resin. Testing showed that both materials performed similarly, with the base resin just edging out the ABS-like material in terms of tensile stress by 1.42 MPa. This equates to just a 6.8% increase in tensile strength for the base resin. As far as strain, both materials showed nearly identical strain rates at 0.075. The main difference between the two resins was that the standard deviation for the ABS-like photopolymer was lower at a value of 1.636 MPa compared to the base resin standard deviation of 2.856 MPa. This increase in deviation was likely the cause for the slightly higher average tensile stress seen in the resin samples.

#### 3.1.2. Analysis of Base Resin Nanocomposites

**Figure 3:**
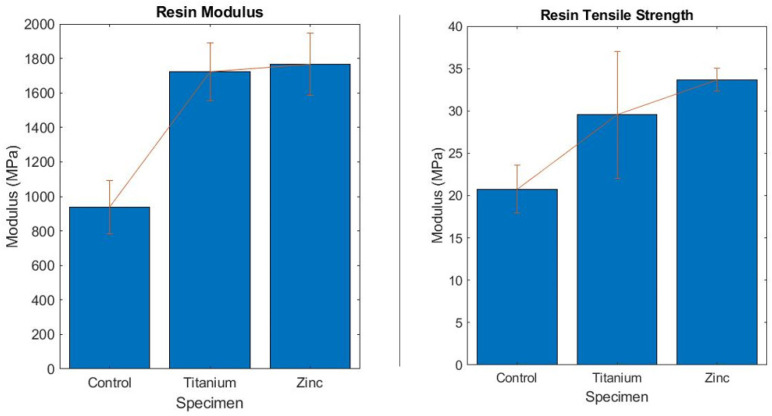
Young’s modulus and ultimate tensile strength bar graphs comparing the resin control and nanocomposites.

The 3D printed nanocomposites with 1 wt% nanoparticles demonstrated a significant increase in tensile strength and Young’s Modulus over the control. Both the ZnO and TiO_2_ had a positive effect on the material’s physical tensile strength. The ZnO led to an average tensile stress of 33.696 MPa, and the TiO_2_ led to an average tensile stress of 29.533 MPa. Along with this, the average strain reduced significantly in both samples. Up to an 88% increase in modulus was observed for the ZnO specimens as depicted in Figure 3. The ZnO sample was reduced to a strain rate of 0.036 and the TiO_2_ sample reduced to 0.026. Thus, the base resin material was a strong candidate for a nanocomposite matrix as the tensile strength increased by 42.2% and the strain reduced by at least 50%.

#### 3.1.3. Analysis of ABS Nanocomposites

**Figure 4:**
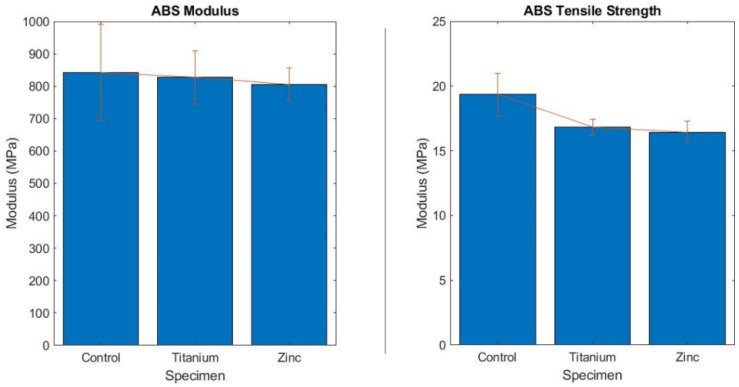
Young’s modulus and ultimate tensile strength bar graphs comparing the ABS control and nanocomposites.

The ABS-like resin showed a negative correlation with the addition of nanoparticles. Both the ZnO and TiO_2_ decreased the tensile strength of the specimens as depicted in Figure 4. The ZnO led to a 15.1% decrease in tensile strength, while the TiO_2_ led to a 12.98% decrease. The modulus reduced by 1.7% for the titanium samples and 4.4% for the zinc. The strain reduction was only 41% for the ZnO and 42% for the TiO_2_. One improvement of the novel nanocomposite was reducing the standard deviation of between 1.03 MPa and 0.81 MPa compared to the ABS-like resin. The reduction in the Young’s modulus and tensile strength in the ABS resin could have been caused by the ununiform dispersion of nanoparticles. Since the ABS resin had an increased viscosity, the dispersion difficulty of nanoparticles in ABS increased dramatically. An improved nanoparticle dispersion can lead to improved mechanical properties in novel nanocomposites.

### 3.2. Abrasion Testing

**Figure 5:**
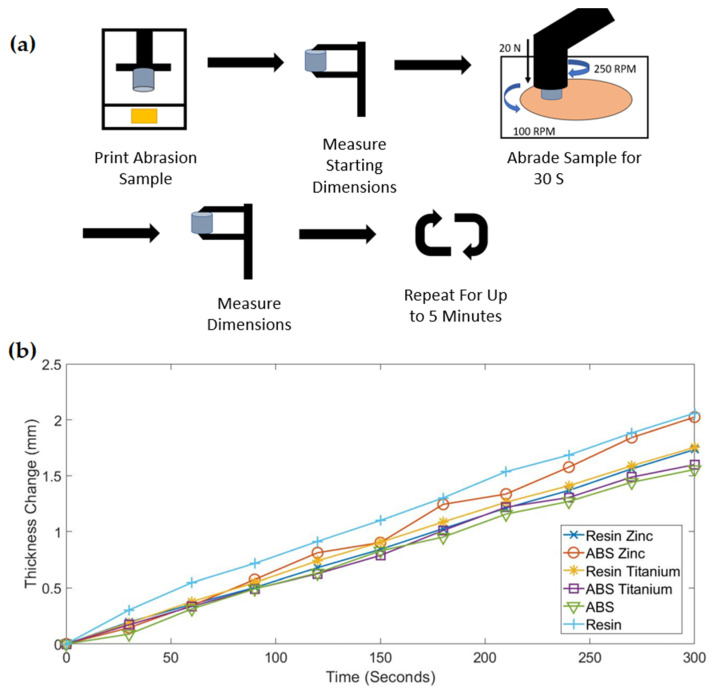
(**a**) Abrasion testing procedure for all samples. (**b**) Abrasion testing results compared across 5 min of abrasion.

The abrasion testing was conducted under a constant water stream and at a pressure of 15.9 KPa. The grinding disc was aluminum oxide with 50-micron-sized abrasives. The disc was rotated at a speed of 100 rpm and the head that held the sample was rotated at 250 rpm. Each sample was put through the grinding process for 5 min at 30 s intervals. The procedure is depicted in Figure 5a.

Starting with the control resins, the ABS-like resin had the least material degradation of all samples at just 1.55 mm. Comparatively, the non-ABS resin had the highest amount of material degradation at 2.059 mm. These tests are equivalent to dragging a part over 471 m of abrasive material with 15.9 KPa of pressure. This equated to over 300 m of abrasion per millimeter of thickness change for the ABS-like photopolymer and over 225 m for the resin.

The nanocomposite samples fell between the above two base samples, with the most notable distinction being the added abrasion resistance in the resin samples. Both resin nanocomposites performed very similarly with a thickness reduction of 1.733 mm for the ZnO resin nanocomposite and 1.752 mm for the TiO_2_ resin nanocomposite. These samples showed a 15.8% increase in abrasion resistance compared to the non-modified material. In contrast, both ABS-like resin nanocomposites showed a decrease in abrasion resistance. The ABS ZnO nanocomposite performed second worst with a thickness change of 2.024 mm. The ABS TiO_2_ sample showed a degradation of 1.598 mm, which was still slightly better than all other non-ABS tests.

### 3.3. Water Contact Angles and Water Absorption

**Figure 6:**
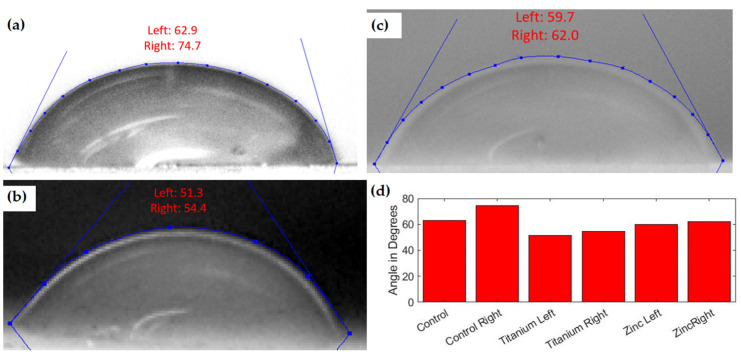
(**a**) Control resin water contact angles. (**b**) TiO_2_ resin nanocomposite water contact angles. (**c**) ZnO resin nanocomposite water contact angles. (**d**) Bar graph of all resin water contact angles.

The last two physical tests performed were water contact angle tests and water absorption tests. These tests were performed to characterize the material’s ability to be used in wet environments for extended periods. The water contact angle measurements were completed for all six specimens using ImageJ software’s drop snake analysis [30]. The factory resin showed an average contact angle of 68.8 degrees. All water contact angle tests were performed on the printed surface with no alterations performed post-printing. The TiO_2_ was observed to reduce the water contact angle to an average of 52.85 degrees. This reduction was also seen in the ZnO nanocomposite but to a lesser degree, achieving an average contact angle of 60.85. As shown in Figure 6, the titanium nanocomposite had the lowest water contact angle across all tests for the resin matrix.

**Figure 7:**
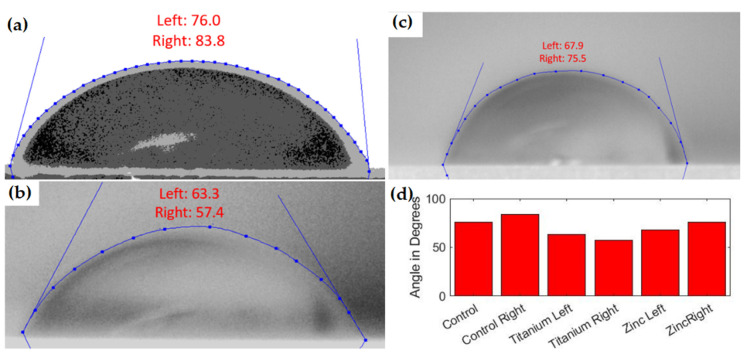
(**a**) Control ABS water contact angles. (**b**) TiO_2_ ABS nanocomposite water contact angles. (**c**) ZnO ABS nanocomposite water contact angles. (**d**) Bar graph of all ABS water contact angles.

The ABS matrix performed similarly but with higher overall values for all tests. The control contact angle was 16.1% higher at an average of 79.9 degrees. This larger contact angle was observed to affect nanocomposites similarly with average contact angles of 60.5 and 71.7 degrees for TiO_2_ and ZnO nanocomposites, respectively. As observed in the resin specimens, the TiO_2_ also demonstrated the lowest water contact angle overall for the ABS matrix material, with all data observed shown in Figure 7.

**Figure 8:**
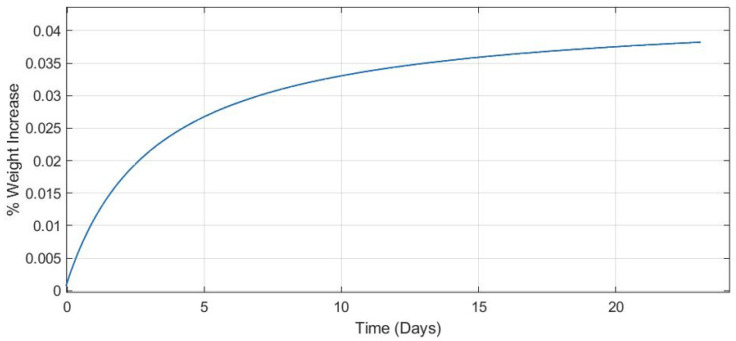
Water absorption percentage over time.

Since the parts demonstrate hydrophobic properties, it was vital to ensure that the water absorption of the material was relatively low to ensure that they could still be applicable in wet environments. Water absorption testing performed followed ASTM D570 for plastics. The tests were performed for over 20 days and the maximum water absorption rate recorded was 4% by weight—the absorption rate plateaued at day 15 following a logarithmic curve as shown in Figure 8. Within the first 24 h, the samples saw a 1.39% gain in weight and a 1.73% gain over the first seven days. The specimens were checked after six months of continued submersion and saw no increase past the 4% observed in the first 15 days. Specimens were left at room temperature for all tests.

### 3.4. SEM Analysis

**Figure 9:**
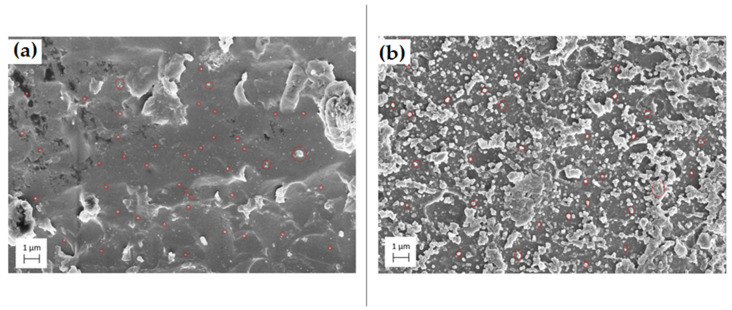
(**a**) SEM imagery of TiO_2_ particles in a resin sample. (**b**) SEM imagery of ZnO particles in a resin sample.

SEM provided a validation of the homogenous dispersion across the surface of the printed pieces. An even dispersion with little clumping was required to observe antibacterial traits on the surface of the prints. The SEM images provided in Figure 9 show TiO_2_ and ZnO on the surface of the resin matrix. Both images were taken from parts produced on the printer with no modification except for sputter coating, which was required for the SEM process. The nanoparticles can be seen circled in red on each of the below images. Again, minimal clumping was observed, and the dispersion was excellent across the entire inspected image.

## 4. Discussion

### 4.1. Three-Dimensional Printing Applications

The critical discovery within this work was the rapid synthesis and production of antibacterial parts using 3D printing technologies. The ability for non-specialists to produce custom parts in under several hours specifically designed for the medical field will help speed up the adoption of 3D printing into the medical sector. The materials used were easy to handle and store while only requiring basic safety measures such as nitrile gloves and eye protection. Therefore, hospitals will not require specialized training and facilities for implementation. Photocurable-based AM has already proven to be one of the best-suited technologies for the healthcare industry [31,32,33,34]. LCD-based printing systems also offer a significant advantage in processing speed as entire layers can be cured at once compared to more expensive SLA machines [34]. The most notable example was the prosthetic finger, as all pieces were printed on one build plate.

Due to the high accuracy possible with these machines, it is straightforward to manufacture parts that interface together [35]. The prosthetic finger example can be assembled in mere min and can easily be sized to any individual. The design freedoms available when using AM are widely known, but implementing these machines into end-use cases has been problematic due to the array of downsides associated with this new technology. Photocurable 3D printing paired with novel nanocomposites, as documented here, suffers from very little of the disadvantages that other 3D printing technologies suffer from. The part’s physical properties are entirely suitable for biomedical uses, the accuracy and resolution are well within desired tolerances, and the ease of manufacturing allows for trouble-free manufacturing.

Resins’ unique printing ability allows for a part to be printed with no visible layer lines. A design of a 3D chess rook was printed in an ABS ZnO nanocomposite to showcase the intricate detail that this novel resin can uphold while still being fully functional. As the printing process is performed layer by layer, intricate details such as internal features can be produced, as seen by the internal staircase on the rook. The other benefit is that large hollow cavities can be drained of resin during the washing process and cured empty to reduce the overall weight. This leads to design freedom not abundantly found in composite manufacturing and, most importantly, not easily implemented in traditional manufacturing.

### 4.2. Physical Properties

Tensile testing results have shown two interesting critical differences between the novel nanocomposites. The first noticeable data point was the similarities between the factory resin materials when looking solely at tensile strength. The average tensile strength between the two materials differed by only 7%. Along with this, the strain rate differed by only 0.27%. The standard deviation for the resin was noticeably higher at a value of 2.856, leading to a 95% statistical confidence interval that both sample’s mean tensile stress values were within the same range. These data appeared to show that both factory materials were physically no different when a tensile force was applied.

Analyzing the abrasion data was where the difference between the two materials became apparent. The factory ABS-like photopolymer outperformed all other tested materials and had a 24.7% increase in abrasion resistance compared to the factory resin. This difference constitutes the main design point behind the ABS-like material when printing end-use functional parts. This increase in abrasion resistance also makes the material a strong candidate for implementation where low stresses will be seen, but high traffic is expected.

The addition of the nanoparticles to both matrix resin materials was where the data started to differ drastically. The nanocomposite addition reduced the properties of the ABS-like photopolymer in every test performed, whereas it improved the properties in every test for the resin material. The only exception to this was the water contact angle. As seen in the tensile test Figure 4, the lower physical properties were not caused by anomalies during the printing process as each part failed at similar yield strengths. In addition, the lowest standard deviation of all sample tests was in both ABS nanocomposite resins. This leads to the inference that the ABS-like resin’s chemical makeup was not a strong candidate for nanoparticle addition. The probable cause of this is that during the curing process, the adhesion of the ABS-like resin and the nanoparticle was less than ideal. The lower results could be a side effect of micro-fractures caused by an incomplete bond between the nanoparticle and matrix. The only test where the addition of the nanoparticle had a minimal effect was within the ZnO ABS nanocomposite abrasion sample. The reduction in abrasion was within the margin of error for having the same properties as the factory ABS-like matrix. The ZnO ABS nanocomposite did not follow a linear abrasion profile such as all other samples. There were points during the testing where substantially more or less material was removed. This means that the mixture of the two materials throughout the test pieces was not perfectly homogenous and varied based on layer depth. There could be several reasons for this phenomenon: particle settlement, clumping, and an uneven disruption of the mixture from the build plate. The TiO_2_ ABS-like nanocomposite did not follow this same path and performed far worse than the factory ABS-like resin similar to the tensile tests. Although the titanium did perform worse, it did follow a linear path with respect to time, as expected in an abrasion test. Because of this, a more uniform mixture can be inferred. This was the cause for the higher tensile stress and lower standard deviation seen compared to its ZnO ABS-like counterpart. The physical difference in size between the two nanoparticles was at most 20 nanometers but only about 10–15 nanometers on average. The density difference of 1.38 g/cm^−3^ between the two nanoparticles contributed to the difference in the compatibility with the ABS-like resin. As the ZnO has a higher density, there would be less material added per gram than the TiO_2_, leading to reduced physical attributes.

Although the ABS-like material did not beneficially pair with the nanoparticles, the base resin did. The resin material saw a significant improvement in all physical properties when paired with a nanoparticle and still reattained great printing attributes. When analyzing the tensile testing data, the first noticeable difference was the increased tensile testing strength in the ZnO nanocomposite. When looking at the ABS-like samples, the ZnO nanoparticles performed worse than the TiO_2_, but the opposite appeared to happen here with the base resin. The ZnO showed a 38.4% improvement in tensile strength and a 52% decrease in strain. This result was expected as the nanocomposite created a much more rigid part that could withstand higher stresses as the nanoparticle helped disperse the load. These results reinforce the idea that a superior bond between the base resin and the nanoparticles took place compared to the ABS-like resin. ZnO did not outperform TiO_2_ in abrasion testing, though, as the type of degradation the part experiences will likely not increase from one nanoparticle to the next. The increase in abrasion resistance for both resin nanocomposites was not the only improved property, but also an improvement in abrasion consistency. Out of all abrasion tests performed, the resin nanocomposites demonstrated the most linear degradation curve with respect to time. This further promotes the theory that there was an improved bonding with the base resin and excellent homogeneity.

Water contact testing demonstrated that there was little effect on the surface of the materials. TiO_2_ was used as previous studies have shown that it tends to create hydrophobic surfaces after being exposed to the sun [36]. As all testing was conducted indoors and long-term sun exposure was not tested, these attributes were not observed on our samples. The water absorption testing did produce results as expected for polymer-based materials and would, therefore, not be a barrier for fluid implementation. Further research into surface modification through sun exposure of TiO_2_ specimens would help provide more supporting evidence for fluid system involvement of parts.

Finally, SEM provided adequate proof that a sufficient dispersion was achieved with minimal clumping. As these parts were printed layer by layer, the SEM imagery showed the first and last layer of the print depending on the side it was taken. This reinforces the data that particle settlement was not an issue during the printing process. This also showed that new nanoparticles will be continuously exposed to the surface as the part wears down to keep the part’s antibacterial properties active.

## 5. Conclusions

This research portrayed the discovery and possible implementation of an antibacterial nanocomposite resin material that can be implemented in standard LCD printing systems. The novel nanocomposite synthesized improved the physical and chemical properties of the base resin. All materials used were easily sourced and can be manufactured without the use of expensive machinery. In addition, the parts were easily printable on a system that allowed for more design freedom than previously seen in traditional manufacturing processes. Multiple complex parts were produced to provide evidence of different use cases. With the published research of the antibacterial effects of the nanoparticles used and the success of the trials documented within, this process could be effectively implemented in many healthcare industry sectors.

The utilization of two different nanoparticles helped demonstrate a factory resins ability to act as a matrix material across differing compounds. In addition, both nanoparticles have demonstrated antibacterial properties, so they were selected as possible reinforcements. Throughout the testing, ZnO was analyzed to be the best reinforcement material as it led to a more significant improvement in mechanical tests. The TiO_2_ nanocomposite showed a 43.5 MPa decrease in modulus and a 4.2 MPa decrease in ultimate tensile stress compared to its ZnO counterpart. Using TiO_2_ would be beneficial if looking for improved water resistance in outdoor settings.

This new nanocomposite allows for a completely new manufacturing design philosophy as it is now compatible with many AM machines. Utilizing ZnO and TiO_2_ in the 10–30 nm range ensures a proper dispersion along with providing antibacterial traits. It was discovered that the unmodified base resin material was the best matrix for nanoparticle addition, with the ZnO resin providing maximum tensile strength of 33.7 MPa and Young’s Modulus of 1766.8 MPa. This nanocomposite showed improved results in tensile testing, abrasion testing, and water contact angles. In addition, it was able to be 3D printed with excellent tolerances and high reliability. These results lead to the observation that a ZnO resin nanocomposite at a 1% addition by weight would be the best choice for photocurable AM.

## Data Availability

The data presented in this study are available on request from the corresponding author.
